# Peronosporomycetes (Oomycota) from a Middle Permian Permineralised Peat within the Bainmedart Coal Measures, Prince Charles Mountains, Antarctica

**DOI:** 10.1371/journal.pone.0070707

**Published:** 2013-08-02

**Authors:** Ben J. Slater, Stephen McLoughlin, Jason Hilton

**Affiliations:** 1 School of Geography, Earth and Environmental Sciences, University of Birmingham, Edgbaston, Birmingham, United Kingdom; 2 Department of Paleobiology, Swedish Museum of Natural History, Stockholm, Sweden; Institut de Biologia Evolutiva - Universitat Pompeu Fabra, Spain

## Abstract

The fossil record of Peronosporomycetes (water moulds) is rather sparse, though their distinctive ornamentation means they are probably better reported than some true fungal groups. Here we describe a rare Palaeozoic occurrence of this group from a Guadalupian (Middle Permian) silicified peat deposit in the Bainmedart Coal Measures, Prince Charles Mountains, Antarctica. Specimens are numerous and comprise two morphologically distinct kinds of ornamented oogonia, of which some are attached to hyphae by a septum. *Combresomyces caespitosus* sp. nov. consists of spherical oogonia bearing densely spaced, long, hollow, slender, conical papillae with multiple sharply pointed, strongly divergent, apical branches that commonly form a pseudoreticulate pattern under optical microscopy. The oogonia are attached to a parental hypha by a short truncated stalk with a single septum. *Combresomyces rarus* sp. nov. consists of spherical oogonia bearing widely spaced, hollow, broad, conical papillae that terminate in a single bifurcation producing a pair of acutely divergent sharply pointed branches. The oogonium bears a short truncate extension where it attaches to the parental hypha. We propose that similarities in oogonium shape, size, spine morphology and hyphal attachment between the Permian forms from the Prince Charles Mountains and other reported Peronosporomycetes from Devonian to Triassic strata at widely separated localities elsewhere in the world delimit an extinct but once cosmopolitan Palaeozoic to early Mesozoic branch of the peronosporomycete clade. We name this order Combresomycetales and note that it played an important role in late Palaeozoic and early Mesozoic peatland ecosystems worldwide.

## Introduction

The Peronosporomycetes (also known as Oomycota or water moulds) are a class of organisms belonging to the phylum Heterokontophyta, which also includes autotrophs such as diatoms and brown algae [1]–[3]. Due to superficial similarities in their filamentous morphology, spore-like oogonia (egg-containing sacs) and life habits, the Peronosporomycetes have in the past been grouped with the true fungi [4]. However, they can be distinguished by the morphology of the motile stage of their life cycle, in which the zoospores possess two differently shaped flagella used for propulsion; a lateral whip flagellum and a one-haired tinsel flagellum [5]. Peronosporomycetes differ fundamentally from true fungi on a cellular level since their cell walls are composed of cellulose and hydroxyproline as opposed to chitin [4], [5], and the cell nuclei contained in the hyphae-like filaments are diploid as opposed to haploid in true fungi [4].

Peronosporomycetes are saprotrophs or parasites [6]. Some are major plant and animal pathogens in modern ecosystems that are responsible for well-known plant diseases, such as potato blight (*Phytophthora infestans*), sudden oak death (*Phytophthora ramorum*), blister rusts and downy mildews [4]. They are currently responsible for the widespread larch dieback seen in *Larix decidua* in the UK and northern Europe [4], [7]–[10], major damage to tropical commercial plant species [11], and extensive death of selected plants in temperate forests and heathlands in the Southern Hemisphere [12], [13]. Peronosporomycetes are capable of reproducing both asexually and sexually [4], [14]. Asexual reproduction initiates with the formation of a zoosporangium, from which primary and secondary bi-flagellated motile zoospores are released. When reproducing sexually, the male nuclei are injected directly into the oogonium [14], [15]. Zoospores achieve dispersal by means of flagellar propulsion through water films either in soil pore water or on the surface of plants and can also spread through overland flow into fluvial and lacustrine environments. Dispersal is, therefore, favoured in moist, damp environments where the zoospores gravitate towards chemical attractants released by plants such as amino acids, sugars, ethanol and acetaldehyde [16].

Krings et al. [5] reviewed the fossil record of the Peronosporomycetes and concluded that all the reported occurrences of this group older than Devonian are dubious or inconclusive. Confident Palaeozoic and early Mesozoic records are restricted to a small number of occurrences in Devonian, Carboniferous and Triassic permineralised peats and sinter deposits [4], [17]. These include the Devonian Rhynie Chert [18], [19], Carboniferous coal balls from the lower coal measures of the UK [20]–[22], Upper Mississippian cherts from France [23], the Upper Pennsylvanian Grand-Croix cherts of France [24], and Middle Triassic silicified peats from the Fremouw Peak locality in the Transantarctic Mountains of central Antarctica [17]. Similar unpublished spinose spore-like bodies are also known from the Upper Triassic of Hopen, Svalbard Archipelago, but are attributed to Ascomycetes (C. Strullu and S. McLoughlin unpublished data). Multilayered oogonium-like structures possibly attributable to Peronosporomycetes have also been reported from a Jurassic hot spring deposit in Patagonia, Argentina [25]. Other possible examples of Peronosporomycetes have been documented from amber [26]–[29]. However, it is difficult to confidently resolve the affinity of those examples. It has been suggested that outgrowths from a Lower Pennsylvanian fungal sporocarp from Great Britain could represent an example of saprotrophic Peronosporomycetes [30]. A possible perononsporomycete affinity has been suggested for some acritarchs [5], [31] based on similarities in their morphology to oogonia of some extant water moulds.

Their sparse fossil record is unfortunate for understanding the evolution of feeding guilds and energy flow within terrestrial communities, since the Peronosporomycetes are important decomposers and parasites in modern ecosystems, particularly in damp soils and freshwater habitats [32], and are also present in the marine realm [33]. Peronosporomycetes are also significant in a broader evolutionary context since they are possibly among the earliest differentiated lineages of eukaryotes based on phylogenetic analyses of molecular data (e.g. [31], [34]–[36]).

The Permian cherts (silicified peats) of the Prince Charles Mountains contain a range of microbial elements within a glossopterid- and cordaitalean- (gymnosperm) dominated mire palaeoecosystem [37], [38]. The microbial remains are preserved in exquisite cellular detail and retain morphological characters that are not preserved outside of Konservat-Lagerstätten, fossil-bearing deposits with exceptional fidelity of preservation [39]. Microscopic remains within the permineralised peat include delicate organs such as fungal hyphae, spores, and invertebrate exoskeleton parts [38], [40], [41]. Some of these fragile organs are even found within invertebrate coprolites preserved within the peat matrix or inside plant tissues [42]. Here we describe two new forms of peronosporomycete oogonia from the Toploje Member chert of the Prince Charles Mountains that are distinguished from each other primarily by differences in the length and density of the branched external spines. The addition of Peronosporomycetes to the inventory of preserved elements in the fossil community from the Prince Charles Mountains expands the known biodiversity and trophic guilds of the high-latitude peat-forming forests of the Permian.

### Geological Setting and Stratigraphic Age

**Figure 1:**
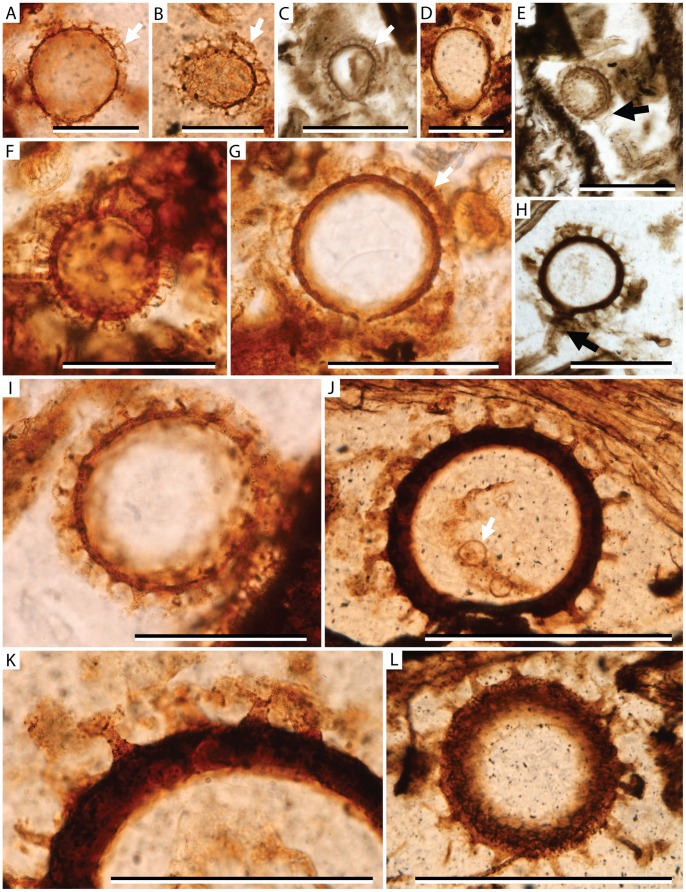
*Combresomyces caespitosus* sp. nov. (Peronosporomycetes: Combresomycetales); Oogonium morphotype with dense coverage of spines. A. NRM S087932-01-02, scale = 50 µm; B. NRM S087800-01, scale = 50 µm; C. NRM S088061-01 oogonium lies within a large coprolitic mass, scale = 100 µm; D. S087800-01, scale = 50 µm; E. NRM S088072-01, arrow indicates position of a possible fragment of hypha extending from oogonium, scale = 100 µm; F. S087800-01, scale = 50 µm; G. S087800-01, scale = 50 µm; H. NRM S087800-01 (holotype specimen), arrow indicates truncate extension with attached subtending hyphae, scale = 100 µm; I. NRM S087932-01-02, scale = 50 µm; J. NRM S087800-01, arrow indicates indeterminate spherical internal contents of oogonium, scale = 100 µm; K. NRM S087800-01, enlargement of wall and ornament of oogonium in image J, scale = 50 µm; L. NRM S087800-01, scale = 100 µm; Arrows in images A–C and G indicate interlocking ornamentation.

Samples of silicified peat were obtained from a 3-km-long outcrop of chert in the northern Prince Charles Mountains, East Antarctica (see Slater et al. [41] fig. 1 for a map of the sampled locality). The silicified interval is ca 40 cm thick and caps a coal seam representing the topmost bed of the Toploje Member within the Bainmedart Coal Measures, the middle unit of the Permo-Triassic Amery Group [43], [44]. The Amery Group is characterised by numerous cycles of thickly bedded sandstones, siltstones and coal seams deposited in an alluvial valley dominated by braided rivers [41], [43], [45]. The cyclicity of the Bainmedart Coal Measures sedimentary facies has been attributed to climatically triggered fluctuations in sediment supply related to Milankovitch cycles [45]. The cause of silicification in the uppermost Toploje Member has not been resolved but appears to be related to geochemical changes in the surface layers of the peat during lacustrine drowning of the mire accompanying deposition of the overlying sideritic–limonitic Dragons Teeth Member [43], [45]. Palynostratigraphic evidence indicates the silicified peat bed is of Roadian to Wordian age [46]. The coals of the lower Bainmedart Coal Measures are of sub-bituminous rank but organic remains within the single layer of silicified peat appear to have been entombed rapidly and shielded from significant compression or thermal alteration [38].

The Amery Group was deposited within a series of half-grabens that constitute the Lambert Graben complex [44], [47], [48], which was a southern extension of the Mahanadi Graben of eastern India before Gondwanan breakup [41], [47], [49]–[51]. The Prince Charles Mountains were situated at a palaeolatitude of 65–70°S during the Middle Permian [52]. The silicified peats, therefore, provide a snapshot of a high-latitude mire community that was likely subject to significant seasonal variation in environmental parameters.

The taphonomy of the silicified peat layer was discussed in detail by Slater et al. [41]. The peat includes a range of plant remains dominated by *Glossopteris* and *Noeggerathiopsis* (glossopterid and cordaitalean gymnosperms, respectively), herbaceous lycophytes and ferns. The community likely represents a raised (ombrotrophic) forest-mire ecosystem based on the substantial thickness of individual coal seams in the Bainmedart Coal Measures, together with the occurrence in the coal of significant quantities of charcoal, fungi and coprolites [42] but minimal siliciclastic components [41].

## Materials and Methods

### Ethics

All necessary permits were obtained for the described study from the Australian Antarctic Division and Australian National Antarctic Research Expeditions program, which complied with all relevant regulations. Specimens are held in the palaeobotannical collections at the Swedish Museum of Natural History (Naturhistoriska riksmuseet), Frescativägen 40, 114 18, Stockholm. Specimen numbers of the material described in this study are; NRM S097800-01, NRM S087932-01, NRM S087932-01-02, NRM S088053-01, NRM S088061-01, NRM S088072-01.

Thirty-five thin sections of the chert were prepared for the analysis of diminutive components of the peats because this method has been shown to reveal greater optical detail of many microbial components than obtainable using the acetate peel technique (see [53]). Images were processed and figures compiled using Adobe Photoshop and Illustrator CS4 graphics packages. Peronosporomycetes was reclassified by Dick et al. [54], however it is important to note that the alternative names for this clade (Oomycetes and Oomycota) are in common circulation in the scientific literature [1], [17]. Although these organisms are not true fungi, their morphological features are still described using mycological terminology. Therefore, this report will describe the hyphae-like filaments as hyphae for consistency with other current literature.

### Nomenclature

The electronic version of this article in Portable Document Format (PDF) in a work with an ISSN or ISBN will represent a published work according to the International Code of Nomenclature for algae, fungi, and plants, and hence the new names contained in the electronic publication of a *PLOS ONE* article are effectively published under that Code from the electronic edition alone, so there is no longer any need to provide printed copies. In addition, new names contained in this work have been submitted to MycoBank from where they will be made available to the Global Names Index. The unique MycoBank number can be resolved and the associated information viewed through any standard web browser by appending the MycoBank number contained in this publication to the prefix http://www.mycobank.org/MycoTaxo.aspx?Link=T&Rec=. The online version of this work is archived and available from the following digital repositories: PubMed Central, LOCKSS.

### Results: Systematic Palaeontology

Kingdom Straminipila M.W. Dick, 2001[a] [4]

Phylum Heterokonta Cavalier-Smith, 1986 [55]

Subphylum Peronosporomycotina M.W. Dick, 2001[a] [4]

Class Peronosporomycetes M.W. Dick, 2001[a] [4]

Order Combresomycetales order nov. B. J. Slater, S. McLoughlin et J. Hilton, 2013

MycoBank number: 804720

### Diagnosis

Peronosporomycetes with oogonia having robust ancyrate sculptural elements.

### Remarks

The new order differs from other groups of equivalent rank in the Peronosporomycetes by the thick wall and robust ancyrate sculptural elements on the oogonia. Oogonia of the Peronosporales and Pythiales may have punctate, papillate, verrucate or simple spinose ornamentation, but none is known to have complex branched sculptural elements [56]. The new order contains a single extinct family diagnosed below.

Family Combresomycetaceae fam. nov. B. J. Slater, S. McLoughlin et J. Hilton, 2013

MycoBank number: 804721.

### Diagnosis

Combresomycetales with oogonia having one to two orders of terminal branching on the conical sculptural elements.

### Etymology for Order and Family

Derived from the type genus *Combresomyces*.

Genus *Combresomyces* Dotzler N, Krings M, Agerer R, Galtier J et Taylor TN 2008 [57].

### Type Species

*Combresomyces cornifer* Dotzler N, Krings M, Agerer R, Galtier J et Taylor TN 2008 [57]; upper Viséan; central France.

*Combresomyces caespitosus* sp. nov. B. J. Slater, S. McLoughlin et J. Hilton.

### Holotype

NRM S087800-01 (Figure 1, image H).

### Type Locality, Stratum and Age

Grid reference 70°49′19″S, 68°03′54″E (elevation 162 m), 1.4 km east of Radok Lake, northern Prince Charles Mountains, Antarctica; uppermost Toploje Member, Bainmedart Coal Measures; Middle Permian (Roadian to Wordian).

### Etymology

Latin – tufted or clumped; referring to the tufted branches that cap papillae.

MycoBank number: 803924.

### Diagnosis

Spherical oogonia having a main body <95 µm in diameter, bearing 6–20 µm long, hollow, slender, conical papillae with at least two orders of strongly divergent, sharply pointed, apical branches. Oogonium attached via a short stalk with single septum to parent hypha 21 µm wide. Papillae spaced 10–20 µm apart.

### Description

The oogonia are spherical with a surface ornamentation consisting of hollow, regularly and densely spaced papillae, which bifurcate at least twice to form a multi-branched terminal crown (Figure 1; Images A–L). This ornamentation interlocks to give the impression of a reticulum in light microscopic examination of some specimens (Figure 1; Images A–C, G). The main body of the oogonium is 40–95 µm in diameter. The wall is 4–15 µm thick. Sculptural elements (papillae and their crowns) are 5–7 µm in basal width and 6–20 µm tall, of which 3–10 µm is the branched crown. The papillae apices bifurcate twice typically and have sharp tips. Papillae are spaced 10–20 µm apart.

None of the specimens demonstrates a connection to a widespread network of aseptate hyphae that is present in the peat, although one specimen is connected to a 32 µm long solitary parental hypha. This parental hypha is 21 µm wide and attaches via a septum to a truncate basal extension of the main oogonium body (Figure 1; Image H). Other oogonia have truncate extensions or breaks in the ornament where the parental hypha presumably attached. It is difficult to discern whether the oogonia have any preserved contents; some specimens house indistinct structures that may represent degraded oospores (Figure 1; Image J) but their identity is inconclusive. Antheridia have not been conclusively identified.

### Remarks

This form occurs dispersed throughout the silicified peat matrix in approximately 50% of the studied thin-sections. It is particularly found in association with accumulations of plant debris around *Vertebraria* (glossopterid roots) and matted leaf deposits of *Glossopteris* and *Noeggerathiopsis*.

Although known to have a conservative morphology spanning the Pennsylvanian to Middle Triassic [17], *Combresomyces* oogonia show subtle differences in size and ornamentation between assemblages of different stratigraphic age. *Combresomyces caespitosus* sp. nov. differs from *Combresomyces cornifer* [57] and *Combresomyces williamsonii* [58] in several respects including slightly denser ornamentation, which abuts or interlocks to form a pseudo reticulum. This feature is not seen in either *C. cornifer* [57] or *C. williamsonii* [58], in which the tips of the ornament remain widely spaced. The oogonia of *C. caespitosus* are larger than specimens of *C. cornifer* from the Pennsylvanian (<40 µm in diameter: [57]) but smaller than the large oogonia of *C. cornifer* known from the Middle Triassic (up to 110 µm in diameter: [17]). The wall of the oogonium in *C. caespitosus* is generally thicker (4–15 µm) than that of both *C. cornifer* (described as *ca* 1 µm [57]) or *C. williamsonii* (described as thin-walled [58]), and the truncate attachment to the parental hypha in *Combresomyces caespitosus* sp. nov. protrudes further from the main body of the oogonium.

The dense multi-branched spines of *Combresomyces caespitosus* oogonia, though markedly smaller, show remarkable similarities in basic morphology to the branched ornamentation of some lycophyte megaspores found in the same beds (see *Singhisporites hystrix* [41]). These similarities might be due functional parallels between these organs as biological dispersive units. The increased surface area generated by densely ramified appendages might have conferred improved buoyancy for dispersal of both megaspores and oogonia in Permian wetland settings [17], or have provided a favourable mechanism for attachment of these structures to other materials (e.g., plant debris in the case of the peronosporomycete saprotroph, or conspecific microspores in the case of the lycophyte megaspores, or even attachment to arthropod distributers). Surface sculptures of a broadly similar morphology occur in many unrelated groups and likely performed an important biological function in life (e.g. [59]).

*Combresomyces rarus* sp. nov. B. J. Slater, S. McLoughlin et J. Hilton.

### Holotype

**Figure 2:**
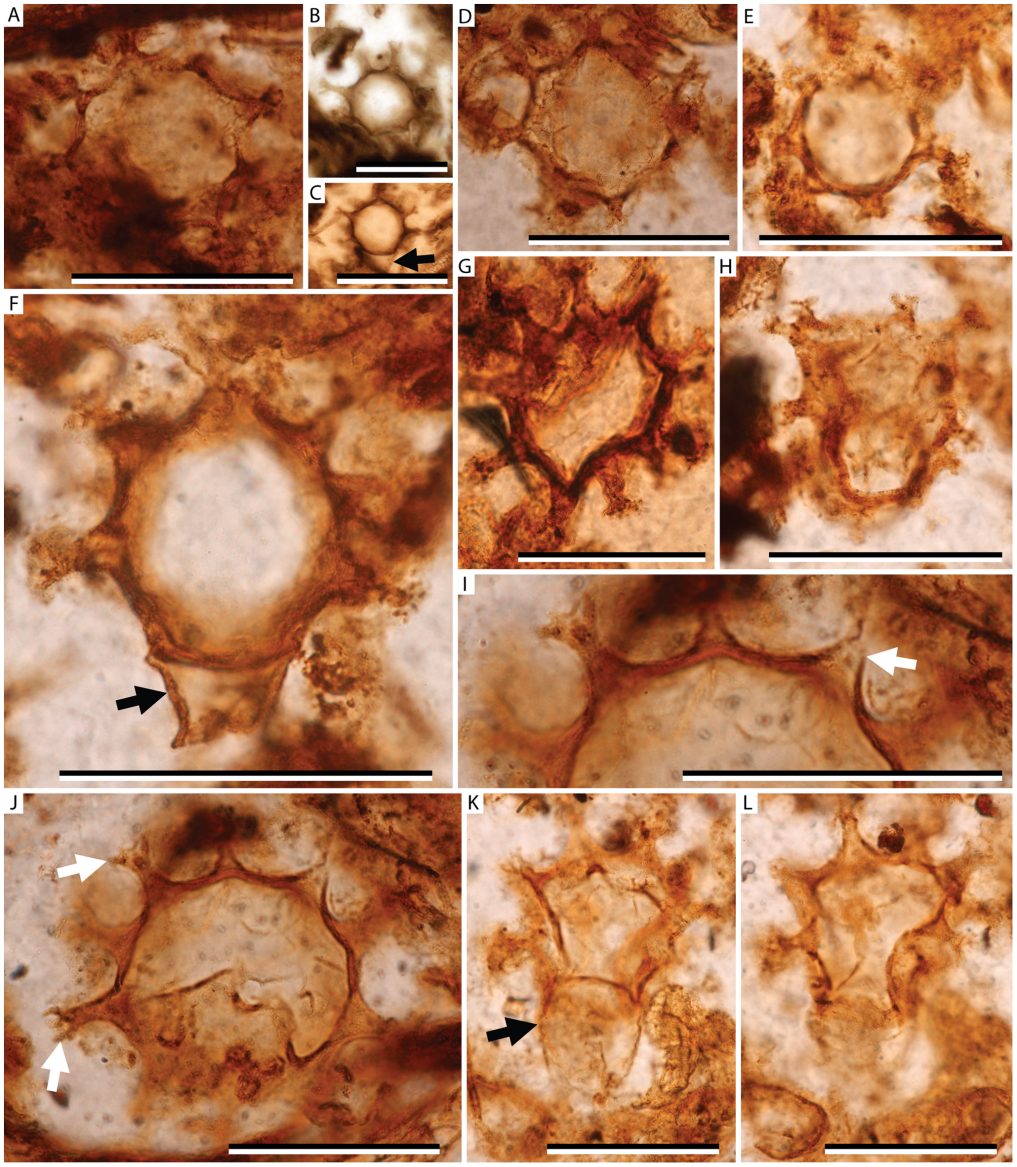
*Combresomyces rarus* sp. nov. (Peronosporomycetes: Combresomycetales); scale bars = 100 µm unless stated otherwise. Oogonium morphotype with sparse coverage of spines; some oogonia contain indeterminate contents. A. NRM S087932-01-02; B. NRM S087932-01; C. NRM S088053-01 arrow indicates attachment point to hyphae; D. S087932-01-02; E. S087932-01-02; F. NRM S087932-01-02 (holotype specimen) arrow indicates attached subtending hypha; G. NRM S087800-01; H. NRM S087932-01-02; I. NRM S087932-01-02 enlargement of oogonia wall and ornamentation, arrow indicates the hollow nature of the papillae, scale = 50 µm; J. NRM S087932-01-02 arrows indicate branched crown of ornamentation, enlargement shown in image I, scale = 50 µm; K. NRM S087932-01-02 arrow indicates attached subtending hypha; L. NRM S087932-01-02 oogonium in image K in different focal plane highlighting the nature of the ornamentation covering the oogonium surface.

NRM S087932-01-02 (Figure 2, image F).

### Type Locality, Stratum and Age

Grid reference 70°49′54″S, 68°03′05″E (elevation 166 m), 1.25 km east of Radok Lake, northern Prince Charles Mountains, Antarctica; uppermost Toploje Member, Bainmedart Coal Measures; Middle Permian (Roadian to Wordian).

### Etymology

Latin – sparse; referring to the widely spaced papillae.

MycoBank number: 803925.

### Diagnosis

Spherical oogonia having a main body <65 µm in diameter, bearing 12–20 µm long, hollow, broad, conical papillae that terminate in at least one bifurcation producing a pair of, generally acutely divergent, sharply pointed branches. Oogonium wall locally bearing a short truncate extension marking attachment point to parent hypha. Papillae spaced 15–20 µm apart.

### Description

The oogonia are spherical with sparsely ornamented surfaces. The ornamentation consists of widely spaced, robust, hollow papillae with elongate extensions that bifurcate at least once (Figure 2). The oogonium has a central body 38–65 µm in diameter with a wall 2–5 µm thick. The sculptural elements (papillae and apical spines) are 12–20 µm in total length, of which 5–8 µm represents the apical branches. Branch apices are sharply pointed. Papillae are 5–8 µm in basal width and spaced 15–20 µm apart – their bases being confluent to produce intervening broad U-shaped transverse sections of the oogonial wall.

None of the specimens demonstrates a connection to a widespread network of aseptate hyphae found within the peat matrix although some were found connected to short lengths of solitary parental hypha (Figure 2; Images C, F, K). The oogonia of the holotype specimen is connected to a 22 µm long length of solitary parental hypha. The parental hypha is 35 µm wide and attaches via a septum to a truncate basal extension of the main oogonium body (Figure 2; Image F). A truncate extension marked by a break in ornamentation is evident on some specimens (Figure 2; Images B and H) and is interpreted to be the attachment point between the oogonium and the parent hypha. The contents of the oogonia are difficult to elucidate. Antheridia have not been identified although it is notable that the length of hypha in one specimen (Figure 2; Image K) is somewhat morphologically similar to the outline expected if the antheridium was formed as a collar-like structure at the base of the oogonium in an amphigynous arrangement. However, the preservation is insufficient to confirm or refute this and we interpret the structure as a length of parental hypha.

### Remarks

This species is less abundant than *Combresomyces caespitosus* in the Toploje Member chert fossil ecosystem, occurring in approximately 25% of the thin sections prepared from the peats. This form occurs primarily in association with *Vertebraria* and *Australoxylon* (respectively, the root and stem wood of glossopterids). Although of equivalent absolute length, the sculptural elements in *Combresomyces rarus* sp. nov. are proportionally longer in relation to the central body of the oogonium than in *C. caespitosus*, and the ornament does not appear to interlink to form a pseudoreticulum.

The ornament of *C. rarus* is more akin to that of *C. williamsonii* [58] than *C. caespitosus*, although the papillae are much more widely spaced with the bases merging to form broad U-shaped transverse sections of the oogonial wall, a feature not seen in *C. williamsonii* [58]. The ornament of *Combresomyces rarus* is also proportionally larger and less densely distributed than in *C. williamsonii* [58].

## Discussion

We refer these fossils to the Peronosporomycetes based on their morphological similarity to examples of this biological class known from other late Palaeozoic and early Mesozoic assemblages, in which oogonia are more confidently associated with hyphae, e.g., *Combresomyces cornifer* [17] and *C. williamsonii* [58]. Similar isolated oogonia attributed to this group are also known from the Pennsylvanian of France [3]. Our confidence in attributing the fossils to the Peronosporomycetes is enhanced by several specimens (Figure 1; Image H, Figure 2; Images C, F, K) possessing a distinctly truncated extension from the main body of the oogonium that attaches to the parent hypha in the same way as specimens of *Combresomyces cornifer* [17]. This truncated extension is present in some fossil examples of Peronosporomycetes and in most extant forms, although it is absent in some [19].

Acyrate-conate fossil oogonia attributable to Peronosporomycetes vary significantly over their stratigraphic range. Earlier forms, e.g., from the Pennsylvanian [57] are generally <40 µm in diameter, whereas Middle Triassic forms reach 110 µm in diameter [17]. The Prince Charles Mountains examples appear to be the first recorded Permian representatives of this group of Peronosporomycetes and possess distinctive ancyrate-conate oogonia of an intermediate size range (38–95 µm). Schopf [60] figured what was described as; “the spiny spore with septate germinal tube, possibly fungal zygospore” (illustrated in figure J of the Schopf paper [60]), which may be a Permian peronosporomycete, though branched ornamentation is not visible on the original illustration. The apparently sparse fossil record of this group may in part be attributable to the past prevalence of the acetate peel technique in studying permineralised plant assemblages. Acetate peels have been shown to be inferior to thin sections in revealing the microorganisms preserved in silicified deposits [53]. Thin sections provide a greater depth of section (30–50 µm) and reveal greater clarity of characters in a range of fungi, fungi-like organisms and diminutive arthropods with thin-walled tissues than is obtainable with mounted acetate peels of ca 10 µm thickness [53], [61].

Ovoid structures possibly also representing oogonia have been reported attached to specimens of *Galtierella biscalithecae* from the Upper Pennsylvanian Grand-Croix Cherts from France [3]. These are also interpreted to be terminally inserted on the hyphae and but are typically oblong and longer than the oogonia described herein. Possible oogonia have also been reported from the Jurassic San Augustín hot spring deposit from Patagonia, Argentina [25] although they lack the forked spines seen in the Prince Charles Mountains examples.

The oogonia of extant Peronosporomycetes, such as *Phytophthora* [62], form terminally and have a range of morphologies from obpyriform to ellipsoid to ovoid [63]. The two morphologies of peronosporomycetes evident in the Toploje Member peat are both covered in ancyrate conate/spinose ornamentation. Fossil examples of this style of ornamentation are numerous [5], [17], [23], [58]. However, the order/family-level affinity of these bodies remains poorly resolved. Although extant Peronosporomycetes oogonia bear ornamentation, none appears to have complex branched crowns on the sculptural elements, a character which is used to distinguish the new order. Some modern *Pythium* oogonia have robust spinose ornamentation [64], [65], especially those of *P. oligandrum* [66], [67], and *P. prolatum* Hendrix and Campbell [68], but the extant forms typically lack forked apices on the spines. Other extant forms that exhibit broadly similar robust spinose/conate ornamentation include *Aphanomyces stellatus* [69], [70], [71]. Among extant forms, *Pythium prolatum* demonstrates particular similarities with those forms from the Toploje Member peat since it possesses the most heavily ornamented oogonia and has a similar truncate extension adjoining the parental hyphae [68]. It seems likely that the various fossil forms represented by oogonia with truncate or branched papillae (including *Combresomyces, Frankbaronia* and perhaps *Hassiella*, *Galtierella* and some members of *Zygosporites*) represent a widely distributed extinct late Palaeozoic to early Mesozoic clade within the Peronosporomycetes and are here placed in the new order Combresomycetales. This group is distinguished by its apically branched conate to spinose ornamentation on the surface of the oogonia, but known morphological characters are as yet insufficient to infer close a phylogenetic relationship with any one of the extant orders of Peronosporomycetes.

Despite the poor fossil record of this extremely diverse class (attribution of several fossil examples being equivocal due to the difficulty in identifying diagnostic characters), the documentation of the group’s occurrence in palaeocommunities is significant since they are important shapers of modern ecosystems. Ancient Peronosporomycetes, like their modern counterparts, probably played a significant role in recycling organic matter, via saprotrophy, and potentially in parasitizing plants and animals in the Permian high-latitude mire ecosystems. In terms of life habit and ecology, both *Combresomyces caespitosus* sp. nov. and *Combresomyces rarus* sp. nov. appear likely to have occupied a saprotrophic lifestyle. This is based on the association of the oogonia of both species with a broad range of adjacent plant tissues. Neither *C. caespitosus* nor *C. rarus* are consistently associated with any one plant type in the permineralised peats, which suggests they did not have a well-developed parasitic relationship with a particular host species.

The recognition of robustly ancyrate-conate Peronosporomycetes oogonia in Middle Permian silicified peats helps bridge the large (latest Carboniferous to Middle Triassic) gap in the group’s fossil record noted by Schwendemann et al. [17] and attests to the broad climatic tolerance of this group, spanning the palaeotropics to cool palaeotemperate belt; fossil Peronosporomycetes oogonia are known from palaeolatitudes as divergent as the palaeotropics of the Viséan of central France [57], [72] to the high palaeolatitudes (ca 65°–70° S) of the Prince Charles Mountains (this study) based on broadly accepted continental reconstructions [52], [73]. Their broad stratigraphic and palaeoclimatic distribution also suggests that the group as a whole was not tied to particular plant hosts; the host floras from the Carboniferous to Triassic variably being dominated by arborescent lycophytes, glossopterids, corystosperms and conifers [5], [17], [57]. This versatility with respect to plant hosts and their distribution through the late Palaeozoic and into the Triassic indicates that Combresomycetales were generalist or opportunistic organisms that were little affected by the end-Permian biotic crisis [74] and the disappearance of peat-forming ecosystems for over 5 million years during the Early Triassic [75], [76].

### Conclusions

Oogonia with multi-branched sculptural elements do not appear to be represented amongst modern Peronosporomycetes based on our survey of the literature, although published details of oogonia and oospore morphology are admittedly sparse. Nevertheless, the obvious similarities in oogonium shape, size, process morphology and hyphal attachment between the Permian Antarctic forms reported here and fossils documented from the Devonian to Triassic elsewhere in the world suggest that these forms represent an extinct but once widespread Palaeozoic to early Mesozoic branch of the peronosporomycete clade. A new order and family of fossil Peronosporomycetes, Combresomycetales B. J. Slater, S. McLoughlin et J. Hilton and Combresomycetaceae B. J. Slater, S. McLoughlin et J. Hilton, are established on this basis. Two new species of *Combresomyces* are distinguished primarily on subtle differences in oogonium size and ornamentation. These represent the first examples of this group documented from the Permian of Antarctica and add to the biodiversity and trophic levels recognised in high-latitude Permian mire ecosystems of Gondwana. The lack of a consistent association between the oogonia and any particular plant fossils in the permineralised peat or of any reaction tissue in adjacent plant remains suggests that these *Combresomyces* species were saprotrophs rather than parasites. Their complex ornamentation may have been an adaptation for aquatic dispersal or adhesion to host materials in the extensive wetlands of the Gondwanan Permian, yet this group of elaborately sculptured Peronosporomycetes as a whole were sufficiently generalist in their ecology to survive the demise of peat-forming ecosystems during the first five million years of the Triassic.
